# yEvo: experimental evolution in high school classrooms selects for novel mutations that impact clotrimazole resistance in *Saccharomyces cerevisiae*

**DOI:** 10.1093/g3journal/jkac246

**Published:** 2022-09-29

**Authors:** Matthew Bryce Taylor, Ryan Skophammer, Alexa R Warwick, Renee C Geck, Josephine M Boyer, yEvo Students, Margaux Walson, Christopher R L Large, Angela Shang-Mei Hickey, Paul A Rowley, Maitreya J Dunham

**Affiliations:** Department of Genome Sciences, University of Washington, Seattle, WA 98195, USA; Program in Biology, Loras College, Dubuque, IA 52001, USA; Westridge School, Pasadena, CA 91105, USA; Department of Fisheries and Wildlife, Michigan State University, East Lansing, MI 48824, USA; Department of Genome Sciences, University of Washington, Seattle, WA 98195, USA; Department of Biological Sciences, University of Idaho, Moscow, ID 83844, USA; Westridge School, Pasadena, CA 91105, USA; Moscow High School, Moscow, ID 83843, USA; Department of Genome Sciences, University of Washington, Seattle, WA 98195, USA; Department of Genome Sciences, University of Washington, Seattle, WA 98195, USA; UW Molecular and Cellular Biology Program, University of Washington, Seattle, WA 98195, USA; Department of Genome Sciences, University of Washington, Seattle, WA 98195, USA; Present address: Department of Genetics, Stanford University, Biomedical Innovations Building, Palo Alto, CA 94304, USA; Department of Biological Sciences, University of Idaho, Moscow, ID 83844, USA; Department of Genome Sciences, University of Washington, Seattle, WA 98195, USA

**Keywords:** yeast, azole resistance, experimental evolution, genome sequencing, science education, course-based research experience

## Abstract

Antifungal resistance in pathogenic fungi is a growing global health concern. Nonpathogenic laboratory strains of *Saccharomyces cerevisiae* are an important model for studying mechanisms of antifungal resistance that are relevant to understanding the same processes in pathogenic fungi. We have developed a series of laboratory modules in which high school students used experimental evolution to study antifungal resistance by isolating azole-resistant *S. cerevisiae* mutants and examining the genetic basis of resistance. We have sequenced 99 clones from these experiments and found that all possessed mutations previously shown to impact azole resistance, validating our approach. We additionally found recurrent mutations in an mRNA degradation pathway and an uncharacterized mitochondrial protein (Csf1) that have possible mechanistic connections to azole resistance. The scale of replication in this initiative allowed us to identify candidate epistatic interactions, as evidenced by pairs of mutations that occur in the same clone more frequently than expected by chance (positive epistasis) or less frequently (negative epistasis). We validated one of these pairs, a negative epistatic interaction between gain-of-function mutations in the multidrug resistance transcription factors Pdr1 and Pdr3. This high school–university collaboration can serve as a model for involving members of the broader public in the scientific process to make meaningful discoveries in biomedical research.

## Teaser

We present data from a unique collaborative education-research endeavor we call yEvo, in which students use experimental evolution of yeast to study the genetic basis of antifungal resistance, a growing public health and agricultural problem. In addition to finding mutations in known azole resistance genes, we discovered new genes and tie some of these to possible mechanisms. We additionally provide evidence for epistatic interactions between mutations, which suggest connections between pathways that have not previously been linked to this phenotype.

## Introduction

Azoles are the primary class of antifungals used in medicine and agriculture. Azole resistance in fungi is an emerging problem that is impacting human health and food security (Fisher *et al.* 2018). Characterizing the genetic basis of azole resistance provides an opportunity to predict whether a newly observed clinical isolate will have resistance to commonly utilized antifungals and may reveal candidates for new treatment paradigms (Cowen *et al.* 2009; Usher and Haynes 2019; Song *et al.* 2020).

Like many antifungal drugs, azoles target a component of cell membrane biogenesis. Azoles competitively bind the active site of the enzyme Erg11, preventing a key rate-limiting step in sterol biosynthesis (Veen *et al.* 2003). Ergosterol in yeasts is functionally equivalent to human cholesterol and the production pathway is highly conserved between humans and yeast (Kachroo *et al.* 2015). Like cholesterol, ergosterol is a key component of the cell membrane, influencing its fluidity, permeability, and organization (Dufourc 2008; Hannich *et al.* 2011). Depletion of membrane ergosterol by inhibiting Erg11 leads to the accumulation of toxic intermediates of sterol biosynthesis causing growth arrest, but not cell death (Kelly *et al.* 1995; Allen *et al.* 2015).

Due to the widespread use of azoles as therapeutics and prophylactics, azole drug resistance has been studied in detail in various species of fungi for decades. Azole resistance mutations fall into two overarching classes. The first class compensates for Erg11 inhibition through the increased production of the Erg11 enzyme (to overcome the competitive inhibition by azoles) or by alterations to the Erg11 active site (to prevent enzyme inhibition by azoles). This can be accomplished through *ERG11* gene duplication, altered activity of regulators of *ERG11* expression, or point mutations in the enzyme itself (Berkow and Lockhart 2017). The second class of mutations, referred to as pleiotropic drug resistance or multidrug resistance mutations (Balzi and Goffeau 1991; Gulshan and Moye-Rowley 2007), lead to increased production or activity of efflux pumps such as Pdr5 (Wolfger *et al.* 2001; Kumari *et al.* 2021). This can be accomplished through point mutations in the pleiotropic drug response transcription factors Pdr1 (Balzi 1987) and Pdr3 (Delaveau 1994), loss of *PDR5* repression, or point mutations in *PDR5* itself (Balzi and Goffeau 1991; Gulshan and Moye-Rowley 2007).

Though the budding yeast *Saccharomyces cerevisiae* is an opportunistic pathogen only under rare circumstances (Clemons *et al.* 1994), it shares drug resistance pathways with pathogenic fungi and has been a useful model for clarifying drug resistance mechanisms beyond the major mutations described above (Paul and Moye-Rowley 2014; Demuyser and Van Dijck 2019).

The majority of genetic studies of azole resistance have utilized traditional mutant selection or clinical isolate screening approaches that tend to focus on single, strong-effect mutations. Experimental evolution provides an opportunity for the identification of mutations with a small or background-dependent effect. Previous evolution experiments selecting for azole resistance have demonstrated that this paradigm can identify new resistance factors and can validate candidate secondary antifungals that prevent resistance evolution (e.g. Cowen *et al.* 2000; Anderson *et al.* 2003; Selmecki *et al.* 2009; Hill *et al.* 2013; Boyer *et al.* 2021; Carolus 2021; Ksiezopolska 2021; Ottilie 2022). We anticipated that additional replicates would lead to the identification of novel resistance factors.

The wealth of information available on azole resistance mechanisms and the possibility of identifying new resistance factors make this system particularly attractive for incorporating authentic research into a high school classroom. To leverage this, we developed protocols suitable for the experimental evolution of *S. cerevisiae* to select for azole resistance in high school classrooms as a course-based research experience called yEvo (available at yevo.org; Taylor 2022). These experiments utilized the drug clotrimazole, which can be found in over-the-counter antifungal treatments. We developed partnerships with teachers at two high schools to ensure these protocols were compatible with their classrooms and learning objectives. The laboratory activities explicitly connect evolution to underlying molecular biology in an open-ended inquiry framework and provide a powerful demonstration of how evolution occurs at the molecular level. Connecting these topics is a key goal of the Next Generation Science Standards (NGSS Lead States 2013), a current benchmark for K-12 science education in the United States.

Our pedagogical aims and evaluation will be reported in detail elsewhere (Taylor 2022). In this paper, we characterize 99 evolved clones from these experiments (performed by 203 students) using phenotyping assays and whole-genome sequencing. We were able to isolate clotrimazole-resistant clones as early as two weeks into the evolution protocol, though the majority of experiments reported here were continued for longer, and in some cases for an entire school year (30–34 weeks), which allowed time for multiple mutations to arise in most clones. Evolved clones were enriched for mutations impacting known azole resistance factors, as well as in genes that had not previously been associated with azole resistance. We provide evidence that these mutations impact azole resistance, expanding our understanding of the genetics of this important trait. We further show that epistatic interactions between evolved mutations can impact the evolution of resistance.

## Materials and methods

### Yeast strains

Evolution experiments were performed with lab-derived strains of *Saccharomyces cerevisiae*. Haploid replicates utilized the *MATa* S288C derivative BY4741 and the *MATɑ* S288C derivative BY4742. Five replicates used a diploid S288C derived by mating BY4741 and BY4742. All strain genotypes are listed in Supplementary Table 1 in Supplementary File 1. Strains used in evolution experiments carried a 2 µm plasmid with KanMX, which provides resistance to the general antibiotic G418, and a pigment production pathway that gives each strain a unique color (Supplementary Table 2 in Supplementary File 1; courtesy of the Boeke lab at New York University). G418 was added to the media (200 mg/L) to select for maintenance of these plasmids and reduce the risk of contamination.

### Classroom protocols

#### Westridge School evolution protocol

Evolution experiments were carried out via batch transfer. Every 2–4 days yeast were transferred on a sterile cotton swab from a saturated culture to a tube of fresh media containing clotrimazole. Clotrimazole was obtained from an over-the-counter 1% solution dissolved in 70% isopropanol. Clotrimazole was added directly to culture tubes in the desired concentration prior to the introduction of yeast. Cultures were maintained in 5 mL of YPD medium with 200 mg/mL G418 and clotrimazole without aeration. Most were grown at room temperature, though some were grown at 30°C. Student groups maintained three replicates of an assigned strain throughout their AP Biology course (grades 11–12; their second biology course). Yeast were initially exposed to a low dose of clotrimazole (2.25 µM), which slowed but did not prevent ancestor growth. The dose of clotrimazole was increased at student discretion at 2× intervals (e.g. 4.5, 9, 18 µM, etc.). Cultures were maintained for up to 34 weeks.

#### Moscow high school evolution protocol

**Fig. 1.**
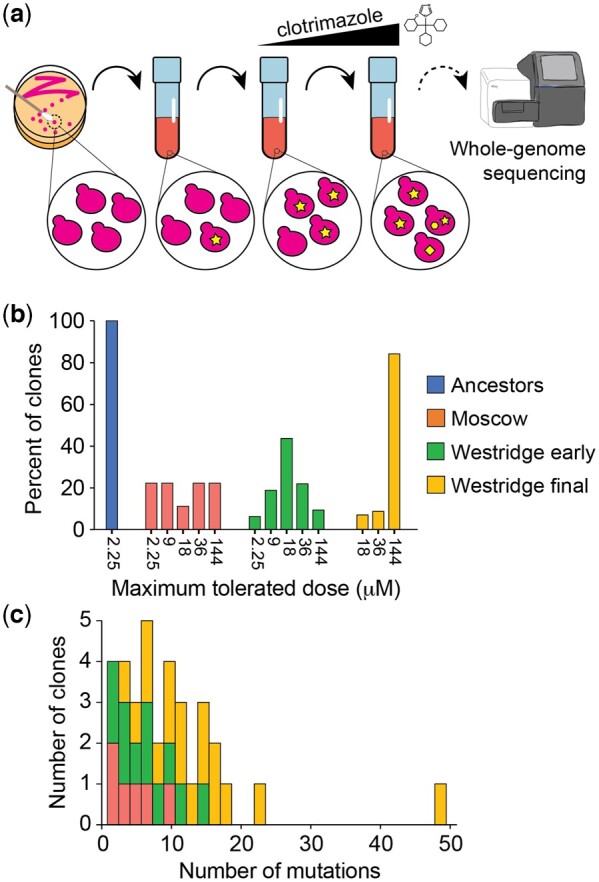
Overview of evolution experiment and sequencing. a) Outline of experiment. Yeasts were propagated in increasing concentrations of clotrimazole for several weeks. Clones from these experiments were sequenced to identify mutations that occurred during the experiment. b) Maximum measured clotrimazole tolerance of clones isolated from experiments at different schools and timepoints. c) Number of point mutations in sequenced clones.

Evolution experiments were carried out via batch transfer. Every seven days, yeast were transferred on a sterile cotton swab from a saturated culture to a tube of fresh media containing clotrimazole. Clotrimazole was obtained from an over-the-counter 1% solution dissolved in 70% isopropanol. Cultures were maintained in 5 mL of YPD medium with G418 (200 mg/L) and clotrimazole at room temperature without aeration. Student groups maintained one replicate of an assigned strain throughout their Honors Biology course (grade 10; first biology course). Yeasts were initially exposed to a much higher dose of clotrimazole (10 µM), which prevented visible growth of the ancestor over short time scales (Fig. 1b), but permitted growth over the extended time between transfers. The dose of clotrimazole was increased at student discretion at 2× intervals (e.g. 20, 40, 80 µM, etc.). Cultures were maintained for up to 12 weeks.

#### Strain storage

Freezer stocks of student populations at Westridge School were generated weekly by adding a 1:1 mixture of 50% glycerol solution in a cryogenic vial and stored in a nonfrost-free −20°C freezer until transfer to −80°C at the University of Washington. Populations from Moscow High School were stored at 4°C until the end of the evolution protocol. Researchers at the University of Idaho isolated clones from these populations by a streak purification method and stored them at −80°C in YPD with 25% glycerol added.

### University lab protocols

#### Phenotyping of evolved clones

Clones from Westridge School were isolated and colony purified from each experiment at two timepoints (early and final, approximately seven and 30–34 weeks, respectively) and at weekly intervals from Moscow High School experiments. One clone was chosen from each student group for whole-genome sequencing. These were assayed to determine their maximum tolerated concentration of clotrimazole as follows: clones were grown for 48 h at 30°C in 200 µL of YPD + G418 medium in a 96-well plate without aeration. Cultures were resuspended, and 2 µL of each culture was transferred to a new 96-well plate containing YPD medium with 200 µg/mL G418 and 2.25, 9, 18, 36, or 144 µM clotrimazole (which correspond to 0.00078, 0.0031, 0.0062, 0.012, or 0.050 mg/L, respectively) and monitored for growth over two days. To assay petite status, clones were additionally transferred to YPG (glycerol as carbon source) + G418 medium and monitored for growth over three days.

#### Whole-genome sequencing

Whole-genome sequencing libraries were generated using a modified version of the Illumina Nextera protocol based on (Baym *et al.* 2015). Briefly, genomic DNA is fragmented using an Illumina Tagmentation enzyme that adds Illumina adaptor sequences to ends. “Tagmented” samples are then PCR amplified using oligonucleotide primers that add unique barcodes to each sample so that samples can be multiplexed on a single Illumina sequencing run. Coverage of all clones described can be found in Supplementary Table 5 in Supplementary File 1. Sequencing reads are deposited in the NCBI Sequence Read Archive (SRA) under BioProject PRJNA742704.

#### Mutation calling

Briefly, reads were aligned to an S288C reference genome using Burrows–Wheeler Aligner (BWA; Li and Durbin 2009) and mutations were called with Samtools. Mutations within 200 bases of a start codon were listed as putative promoter mutations. Single nucleotide polymorphisms with a quality score lower than 100 (out of 228) were discarded. All mutations with a quality score less than 200, as well as all indels, were manually inspected in the Integrative Genomics Viewer (IGV). This was to ensure variants were present in a majority of reads from each mutant and were not present in reads from the respective ancestor. Common mutation targets were manually inspected in IGV to look for evidence of mutations that were missed by our mutation calling pipeline. We identified one such instance in the gene *PDR1* in strain: Westridge_C_final_1_2017-2018.

#### Copy number variants

The average genome-wide coverage was determined in 1,000-bp windows using the program IGVtools and plotted in R. Continuous regions with coverage that exceeded the genome-wide average by ≥2× in haploids were considered increased copy number variants (CNVs). Regions that deviated from the genome-wide average by 0.5× in diploids were considered decreased CNVs. Regions that deviated from the genome-wide average by 1.5× in diploids were considered increased CNVs.

#### Transposable element calling

Clone sequencing data were examined for evidence of mobilization events of transposable elements vs the reference genome using the McClintock pipeline (Nelson *et al.* 2017). Comparisons were made between the ancestral strain and the clones to find exclusively de novo events within our experiments that were at least 1,000-bp away from existing transposon insertions with the software Bedtools. Subsequently, the transposition events were manually inspected in IGV for veracity using the split and discordant reads, generated using BWA, and later processed with SAMblaster (Faust and Hall 2014) and Samtools. One mobilization in the gene *PDR3* was confirmed by PCR and Sanger sequencing using primers PDR3_genotype_F and PDR3_genotype_R (Supplementary Table 3 in Supplementary File 1).

#### Lineage determination

Clones from the same group that shared at least one point mutation were considered as originating from the same lineage. Shared mutations are denoted as nonindependent in Supplementary Table 6 in Supplementary File 1. Mutations that were shared by multiple members of a lineage were considered as single mutation events for the purpose of identifying recurrently mutated genes.

#### CRISPR/Cas9 allele replacements

A PAM (polyspacer adjacent motif) near *CSF1*^A2913P^ was targeted for cutting by the Cas9 enzyme. Oligonucleotides for guide RNA design and repair donors can be found in Supplementary Table 3 in Supplementary File 1. Guide RNA oligonucleotides were introduced into pML104 backbone by Gibson assembly (Supplementary Table 2 in Supplementary File 1; Laughery *et al*. 2015). A mutation substituting G to C was introduced to recreate the *CSF1*^A2913P^ allele detected in one evolved clone. To prevent recutting, a synonymous mutation that altered the PAM was introduced in codon A2913 on its own (control) or in combination with the A2913P mutation. A stationary yeast culture of *MATɑ* haploid S288C (Supplementary Table 1 in Supplementary File 1) was transformed with 100 ng Cas9 and gRNA expression vector (Supplementary Table 2 in Supplementary File 1) and 1 µg donor DNA with the lithium acetate protocol. The genotype of transformed clones at *CSF1* was determined by Sanger sequencing with primers CSF1_genotype_F + CSF1_genotype_R (Supplementary Table 3 in Supplementary File 1).

#### CSF1 allele replacement competitions

Stationary phase cultures of wild-type, a synonymous mutant, and a nonsynonymous mutant were mixed in equal proportions. Fifty microliters of this mixture was inoculated into 5 mL of either YPD or YPD plus 9 µM clotrimazole. These cultures were grown at 30°C in a roller drum until they reached stationary phase after ∼2 days. Fifty microliters of stationary culture was transferred to 5 mL of respective media. This backdiluting was performed twice for a total of three outgrowths. The frequency of *CSF1*^A2913P^ was determined at initial and final timepoints by Sanger sequencing. The frequency of *CSF1*^A2913P^ allele at each sequenced timepoint was determined with the program QSVAnalyzer (Carr *et al.* 2009). Frequencies depicted are averages of three replicates. Error bars are one standard deviation in each direction (Supplementary Table 11 in Supplementary File 1).

#### Tetrad dissections

Diploids were grown overnight in 5 mL YPD in a roller drum at 30°C. One milliliter of stationary-phase culture was pelleted and resuspended in 5 mL sporulation medium as in (Dunham *et al.* 2015). These sporulation cultures were grown on a roller drum at room temperature. After 3–5 days, 50-µL culture was pelleted and resuspended in 15 µL Yeast Lytic Enzyme (YLE) for 22 min at 30°C. Five microliters of water were slowly added to this mixture to dilute cells. Fifty microliters of digested spore mixture were dripped down a YPD agar plate and allowed to dry. Tetrads were dissected with a micromanipulator microscope.

#### Restriction enzyme genotyping

Cleaved Amplified Polymorphic Sequence (CAPS) markers were identified through manual examination of mutant and wild-type sequences in SnapGene. Polymorphisms were amplified with Phusion polymerase with kit protocol. Primer sequences can be found in Supplementary Table 3 in Supplementary File 1. PCR product (8.5 µL) was mixed with 1 µL 10× cutsmart buffer, 0.25 µL enzyme, 0.25 µL water, and digested for 1 h at 37°C. Restriction enzymes RsaI, MboI, HaeIII, BlpI, KpnI, and BamHI were sourced from New England Biolabs.

#### Generation of ERG25^T202P^/ATP2^D382H^ and ERG25^T202P^/ROX1^S46A^ strains

Evolved clones Westridge_W_final_3_2018-2019 (*ERG25^T202P^ ATP2^D382H^ PDR1^E1046G^ HAP1^C84F^ TAO3^A2283P^ PRP5^I779M^ CSG2^M297K^*) and Westridge_W_early_1_2018-2019 (*ERG25^T202P^ ROX1^S46A^ PDR1^G1042E^ PHO4^I117K^ MAK16^A^*^-137T^*KGD1^A930A^*) (Supplementary Table 6 in Supplementary File 1) were each mated to ancestral strain of the opposite mating type YMD895, sporulated, and dissected. Segregants were genotyped and chosen for having fewer unwanted mutations (*ERG25^T202P^ ATP2^D382H^ HAP1^C84F^ TAO3^A2283P^* and *ERG25^T202P^ ROX1^S46A^ PDR1^G1042E^*), then backcrossed again to YMD895 to remove all unwanted mutations and produce combinations of desired mutations. Spores were genotyped by restriction enzyme digestion or Sanger sequencing by Genewiz/Azenta (Supplementary Table 3 in Supplementary File 1) to obtain 3–7 spores per genotype.

#### Plate reader experiments

Genotyped spores were grown in 200 µL YPD medium in 96-well plates for 48 h. Cultures were resuspended and 2 µL of each culture was transferred to 198 µL YPD media with clotrimazole added. The growth of these cultures was monitored in a plate reader for 36–48 h at 30°C with orbital shaking. The average growth rate of all strains with the same genotype was calculated by linear fit to logarithmic growth phase and plotted in R; script is available on GitHub (github.com/reneegeck/DunhamLab/blob/main/platereader_growthplotter.R).

## Results

### Isolation of clotrimazole-resistant clones from a course-based research module

We implemented an experimental evolution lab module at two high schools in the United States. Protocols for these experiments were developed in close collaboration with each teacher to best align with their time, resources, and learning objectives (Fig. 1a; Taylor 2022). Students used a simple serial transfer protocol to evolve yeast populations to grow in inhibitory concentrations of an over the counter antifungal, clotrimazole. All replicates utilized strains that were derived from the standard S288Cc laboratory strain (Supplementary Table 1 in Supplementary File 1). The experiments at each school also differed in several ways (*Materials and Methods*). For instance, students at Westridge School carried out experiments throughout the school year with serial passage occurring every class period, or roughly every 2–4 days. Five replicates from Westridge School utilized diploid strains instead of the haploid strains utilized in all other experiments. Students at Moscow High School carried out experiments for an average of 11 passages at weekly intervals. At the conclusion of both sets of experiments, evolved yeast populations were collected by partnering research laboratories at the University of Washington and the University of Idaho (*Materials and Methods*).

Clones were isolated from evolved populations at early time points (7 weeks at Westridge School and 2–12 weeks at Moscow High School), as well as from later time points (30–34 weeks at Westridge School). These evolved clones were capable of growing in higher concentrations of clotrimazole than the unevolved ancestors, which had a maximum tolerated dose of 2.25 µM (Fig. 1b). Clones from later timepoints were able to grow in high concentrations of azole with 53 of 57 late timepoint clones able to tolerate 144 µM clotrimazole, compared to 14 of 42 early timepoint clones (Fig. 1b; Supplementary Table 4 in Supplementary File 1). Clones from Westridge School tended to have more mutations than clones from Moscow High School, and clones from later timepoints tended to have more mutations than earlier timepoint clones (Fig. 1c). Based on these data, we hereafter refer to these clones as resistant to azoles.

### Whole-genome sequencing of evolved clones identified de novo mutations connected to azole resistance

**Fig. 2.**
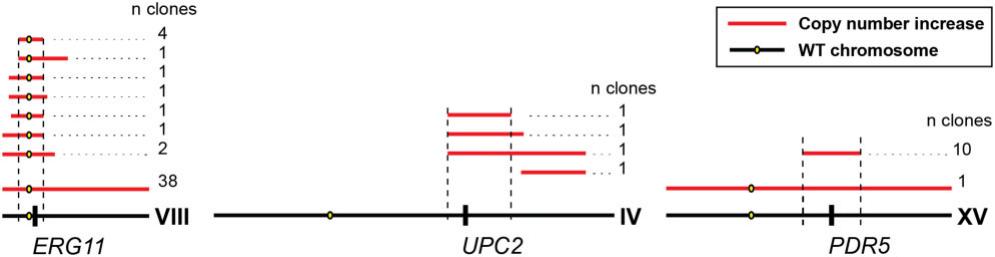
Copy number variation events and candidate genes. Likely segmental duplications on chromosomes VIII, IV, and XV based on increased coverage in whole-genome sequencing data (*Materials and Methods*). Regions with increased copy number in at least one sequenced clone are represented with red horizontal lines drawn above their corresponding chromosomes. A number of clones with a given amplification are listed to the right of each red line. Location of candidate genes that could contribute to a fitness benefit is denoted on wild-type chromosome in black.

The genome sequence of each confirmed drug-resistant clone was determined using short-read next-generation sequencing. The genome sequences of the azole-resistant clones and their ancestors were aligned to the *S. cerevisiae* reference genome to identify sequence differences unique to evolved clones (*Materials and Methods*). We looked for point mutations (Supplementary Table 6 in Supplementary File 1; Table 1), loss of mitochondrial DNA (ρ^0^ petite mutations) (Supplementary Table 8 in Supplementary File 1), copy number changes (Supplementary Table 9 in Supplementary File 1; Fig. 2), and transposable element mobilizations (Supplementary Table 10 in Supplementary File 1; Supplementary Fig. 1a).

**Table 1.** Recurrently mutated genes.

Gene	Unique mutations	No. of clones w/mutation	Function	Literature support for gene?	Literature support for pathway?
*PDR1*	33	65	*PDR5* regulation	Yes (Gulshan and Moye-Rowley 2007; Paul and Moye-Rowley 2014)	Yes (Gulshan and Moye-Rowley 2007; Paul and Moye-Rowley 2014; Kumari *et al.* 2021)
*SUR1*	30	44	Sphingolipid production	Yes (François *et al.* 2009; Kapitzky *et al.* 2010; Vandenbosch *et al.* 2013)	Yes (François *et al.* 2009; Gao *et al.* 2018)
*HAP1*	26	30	Oxygen sensing and ergosterol regulation	Yes (Serratore *et al.* 2018)	Yes (Jordá and Puig 2020)
*ERG25*	25	49	Ergosterol production	Yes (Gachotte *et al.* 1997; Smith *et al.* 2016)	yes (Gachotte *et al.* 1997; Joseph-Horne and Hollomon 1997)
*PDR3*	15	26	*PDR5* regulation	Yes (Gulshan and Moye-Rowley 2007; Paul and Moye-Rowley 2014)	Yes (Gulshan and Moye-Rowley 2007; Paul and Moye-Rowley 2014; Kumari *et al.* 2021)
*CSG2*	13	17	Sphingolipid production	Yes (Kapitzky *et al.* 2010; Vandenbosch *et al.* 2013)	Yes (François *et al.* 2009; Gao *et al.* 2018)
*DHH1*	6	8	mRNA degradation	Yes (Vandenbosch *et al.* 2013)	No
*ROX1*	6	6	Oxygen sensing and ergosterol regulation	Yes (Henry *et al.* 2002)	Yes (Jordá and Puig 2020)
*TAO3*	6^a^	6	RAM signaling	No	Yes (Nelson *et al.* 2003; Song *et al.* 2008; Saputo *et al.* 2012)
*ATP1*	4	7	Mitochondrial ATP synthesis	Yes (Li *et al.* 2017)	Yes (Li *et al.* 2017)
*ATP2*	4	4	Mitochondrial ATP synthesis	No	Yes (Li *et al.* 2017)
*CSF1*	4	5	Unknown	Yes (Mount 2018)	No
*DCP2*	4	5	mRNA degradation	No	No
*SIT4*	4	5	Regulates multidrug resistance and sphingolipid synthesis^b^	Yes (Miranda *et al.* 2010; Vandenbosch *et al.* 2013; Khandelwal *et al.* 2018)	Yes (François *et al.* 2009; Miranda *et al.* 2010; Gao *et al.* 2018)
*HYM1*	3	4	RAM signaling	No	Yes (Nelson *et al.* 2003; Song *et al.* 2008; Saputo *et al.* 2012)
*CBK1*	3	4	RAM signaling	No	Yes (Nelson *et al.* 2003; Song *et al.* 2008; Saputo *et al.* 2012)

Genes with at least three independent nonsynonymous, indel, or nonsense mutations. Literature support is defined by at least one instance in which a gene or pathway has been implicated in resistance or sensitivity to an azole drug or in ergosterol production in a published research article.

a One clone possesses three unique *TAO3* mutations.

b *SIT4* regulates several processes including multidrug resistance and sphingolipid synthesis (Khandelwal *et al.* 2018).

As a quality check on both the evolution procedure and the sequencing analysis, we first looked in our list of genes found to have repeated independent mutations for genes known to confer azole resistance (Table 1; Supplementary Fig. 1, b and c). We found every sequenced clotrimazole-resistant clone had at least one of the following: (1) a nonsynonymous mutation in *PDR1* or *PDR3*, known transcriptional regulators of the pleiotropic drug response; (2) a loss of the mitochondrial genome leading to a petite phenotype, which has also been previously associated with azole resistance; and/or (3) a DNA copy number increase involving *ERG11*, the target of azoles (see below). We then expanded our analysis to more completely catalog all mutations in the evolved clones.

### CNVs were common and may impact azole resistance factors

The majority of clones (61 of 99) had at least one CNV. The most common CNVs involved chromosomes I (7 clones), II (4 clones), IV (4 clones), VIII (49 clones), and XV (11 clones) and were most often full chromosome amplifications or segmental duplications (Fig. 2; Supplementary Table 9 in Supplementary File 1). In addition, two of the five sequenced diploid clones had decreased coverage of chromosome I, indicating a loss of one copy of that chromosome. *ERG11* is on *S. cerevisiae* chromosome VIII, the most frequently amplified chromosome across sequenced clones, making this gene an attractive candidate for providing the observed fitness benefit. This result is supported by the fact that all of our segmental duplications include *ERG11*. Candidate resistance genes are included within all segmental duplications in Fig. 2 and Supplementary Table 9 in Supplementary File 1.

### Loss of mitochondrial genome was common in evolved clones

The loss of the mitochondrial genome (aka petite or ρ^0^ status) occurred in 73% (72 of 99) of drug-resistant clones. These observations were made based on an examination of sequence alignments to the reference mitochondrial genome. Petite mutants have been shown to have an increased resistance to azole drugs (Hallstrom and Moye-Rowley 2000; Ferrari *et al.* 2011), with the tradeoff that they are unable to grow using nonfermentable carbon sources. To confirm that these were petite mutants, all clones were cultured using a medium containing glycerol, a nonfermentable carbon source (Supplementary Table 8 in Supplementary File 1). We identified one clone (Westridge_M_final_1_2018-2019) with a wild-type mitochondrial genome that did not grow in glycerol medium, indicating it had lost respiratory activity due to a nuclear mutation. This clone possessed unique nonsynonymous mutations in the genes *ERG25*, *TDA9*, and *VPS501*, as well as a noncoding mutation 276 bases upstream of the gene *SLP1*. None of these genes have previously been associated with respiratory deficiency, though *TDA9* regulates acetate production and may be an appealing candidate (see *Discussion*).

### Transposable element mobilizations biased toward azole resistance factors

We identified only 14 transposable element mobilizations across all 99 sequenced clones. Only one gene had multiple (four) independent mobilization events (all in independent clones) that interrupted its sequence: *SUR1*, an enzyme involved in sphingolipid production, which impacts membrane composition, a known mechanism that influences azole resistance (François *et al.* 2009). Another mobilization event was detected in the 3′ end of *PDR3*, which encodes a transcription factor regulating the pleiotropic drug response. Both of these genes were found to harbor point mutations in other evolved clones (see below).

### Point mutations in well-characterized azole resistance factors

One goal of the evolution experiments was to isolate clones with multiple mutations that could impact azole resistance. Clones from Moscow High School had an average of 4.8 point mutations, Westridge School early clones had 5.0, and late clones had 11.6. One clone, from a late timepoint at Westridge School had a particularly high number of mutations (48, compared to the next highest with 23). Since clones from Westridge School were isolated at multiple time points, some clones shared mutations. We have denoted shared mutations as nonindependent events, which can be found in Supplementary Table 6 in Supplementary File 1.

In total, 575 unique point mutations were detected across all 99 clotrimazole resistant clones. Of these, 466 were nonsynonymous, indel, or nonsense mutations. A GO term enrichment analysis on this subset (using the GO term finder tool at https://yeastgenome.org/goTermFinder last accessed April 12 2021) found clusters of genes related to drug binding, DNA binding, and regulation of metabolic processes, but with diverse cellular functions (Supplementary Table 7 in Supplementary File 1). Even among these nonsynonymous mutations, we anticipate that a nontrivial fraction will be neutral. In other experimental evolution contexts only 20% (Buskirk *et al.* 2017) to 35% (Payen *et al.* 2016) of all mutations found in evolved clones were estimated to be beneficial. To gain further insight into mechanisms of resistance, attention was focused on genes with three or more independent nonsynonymous or nonsense point mutations, since these are more likely to be causative as opposed to passenger mutations, which should be more randomly distributed (Table 1). Mutations in this subset of genes accounted for 33.9% of all detected mutations. Many of these genes (11/16) or their associated pathways (13/16) have well-characterized connections to azole resistance, which we discuss below and summarize in Table 1 and Supplementary Fig. 1c.

Half of the azole-resistant clones (49 of 99) possessed missense mutations in *ERG25*. None of the mutations in *ERG25* in the experiment were clear null alleles (nonsense or frameshift), as expected since ERG25 is essential unless media is supplemented with ergosterol. We also identified mutations in, and copy number increases centered around, *UPC2* and *PDR5* (Fig. 2; Supplementary Table 9 in Supplementary File 1)*. UPC2* encodes a master regulator of ergosterol synthesis and *PDR5* encodes a major drug efflux pump regulated by Pdr1 and Pdr3. Gain-of-function mutations in both these genes have been shown to impact azole resistance (Flowers *et al.* 2012, *UPC2*; The Uniprot Consortium 2021, *PDR5*).

We also detected mutations in genes that impact sphingolipid production such as *SUR1*, *CSG2*, and *SIT4* (Baudry *et al.* 2001; Gulshan and Moye-Rowley 2007; François *et al.* 2009), in addition to the four transposable element mobilizations interrupting *SUR1* (Supplementary Table 10 in Supplementary File 1). Sphingolipids interact with ergosterol to produce lipid rafts and perturbation of sphingolipid levels is thought to impact azole accumulation (François *et al.* 2009). *SIT4* has been shown to impact the expression of the multidrug resistance pathway as well (Miranda *et al.* 2010).

We detected six independent point mutations in *TAO3*, which encodes a component of the conserved RAM kinase signaling network (Nelson *et al.* 2003). In addition, we detected three independent mutations in RAM network genes *HYM1* and *CBK1* and two each in *KIC1* and *SOG2*. To our knowledge, none of these genes have a reported azole phenotype in *S. cerevisiae* (SGD, accessed 2019 August 30). However, zinc finger transcription factor Ace2, which is regulated by the RAM network (specifically by phosphorylation by Cbk1), has been implicated in both increased azole susceptibility (miconazole) and azole resistance (fluconazole; Kapitzky *et al.* 2010; Vandenbosch *et al.* 2013). In *S. cerevisiae*, null mutations of *ACE2* confer an increased azole resistance when yeast are grown on agar media (Kapitzky *et al.* 2010), and a decreased resistance when grown as biofilms (Vandenbosch *et al.* 2013). Also, deletion of *MOB2* of the RAM network has been shown to cause increased susceptibility to conazoles (Guan *et al.* 2020).

Evolved clones were enriched for mutations in the heme-regulated transcription factor genes *HAP1* and *ROX1*. These genes are regulated by oxygen (Kwast *et al.* 1998) and in turn, regulate a variety of cellular processes including expression of genes involved in ergosterol synthesis (Serratore *et al.* 2018). The lab strain we utilized for our evolution experiments (S288C) has a transposable element insertion near the 3′ end of *HAP1* that reduces the functionality of the Hap1 protein (Gaisne *et al.* 1999). Nine of the *HAP1* mutations we detected are frameshift and 16 are nonsense, indicating that further loss-of-function leads to the resistance phenotype. Two of the six detected *ROX1* mutations are an early stop and a single-base deletion leading to a frameshift (Y204* and I39indel), suggesting that these are null mutations. Indeed, deletions of *ROX1* have been shown to increase azole resistance (Henry *et al.* 2002).

We detected four unique missense mutations in each of the genes *ATP1* and *ATP2* (Table 1), which function in the mitochondrial F1F0 ATP synthase (Saltzgaber-Muller *et al.* 1983; Takeda *et al.* 1986). This complex plays an important role in cellular respiration by synthesizing ATP from the electrochemical gradient generated by the electron transport chain (Nilsson and Nielsen 2016), so it is possible that these mutations impact metabolism in a way that is similar to or synergistic with petite status. Indeed, null alleles of *ATP1* have been shown to exhibit a petite phenotype independent of mitochondrial genotype status (Takeda *et al.* 1986), and all of our *ATP1* and *ATP2* mutants lost their mitochondrial genome. Importantly, mutations in components of the F1F0 ATP synthase, including *ATP1*, have been shown to increase the expression of *PDR5* (Zhang and Moye-Rowley 2001).

### Recurrent mutations in mRNA degradation and an uncharacterized mitochondrial protein

In all clotrimazole-resistant isolates, the prevalence of mutations in genes with known roles in drug resistance supported the efficacy of our selection protocols. However, many additional mutations enriched in several genes or pathways have not explicitly been implicated in azole resistance. All nonsynonymous mutations identified in this study were enriched for the processing body (P-body) GO term (Supplementary Table 7 in Supplementary File 1). This includes genes that decap and degrade inactive mRNAs in P-bodies (Sheth and Parker 2003; Wickens and Goldstrohm 2003; Nissan and Parker 2008). Specifically, six mutations were identified in the gene *DHH1* and four in *DCP2* that encode an activator of mRNA decapping and a decapping enzyme, respectively (Nissan and Parker 2008). Two additional mutations were identified in the 5′-3′ exonuclease *XRN1* that degrades uncapped mRNAs (Larimer and Stevens 1990). The majority of other mutations related to P-bodies had an unclear impact on encoded protein function. However, two mutations in the catalytic N-terminal domain, *XRN1*^S1155*^ and *XRN1*^C201indel^, are likely loss-of-function or null mutations based on their location in this crucial domain (Larimer and Stevens 1990). Furthermore, deletions of *XRN1* (Kapitzky *et al.* 2010; Gao *et al.* 2018) and *DHH1* (Vandenbosch *et al.* 2013) have been shown to increase azole resistance in separate genome-wide deletion collection screens.

**Fig. 3.**
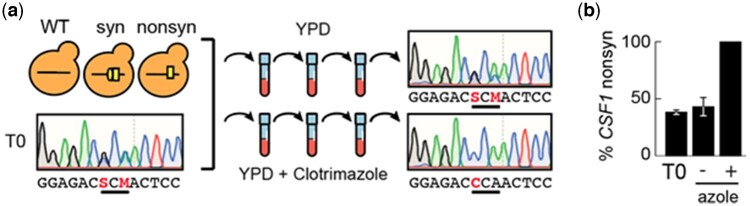
Outline of *CSF1* competition experiment. a) Wild-type, synonymous mutant, and nonsynonymous mutant (*CSF1*^A2913P^) were mixed in equal ratios and inoculated into YPD growth media with or without clotrimazole. These populations were propagated for three outgrowths. The frequency of *CSF1*^A2913P^ was determined at initial and final timepoints by Sanger sequencing. Representative Sanger sequencing chromatograms are shown. Heterozygous positions are represented with IUPAC codes in sequences below chromatograms (S used when G and C present; M used when A and C present). b) Frequency of *CSF1*^A2913P^ allele at beginning and end of competition with (+) or without (−) clotrimazole. Frequencies are averages of three replicates and were quantified by the program QSVAnalyzer. Error bars are one standard deviation in each direction.

The only other gene with four or more mutations that does not have a clear connection to azole resistance is *CSF1*. The function of this gene is unknown, though it has been linked to an inability to ferment at low temperature (Tokai *et al.* 2000) and it is a conserved gene from yeast to humans. To test whether *CSF1* mutations impact azole resistance, we introduced one of these mutations (A2913P) into a wild-type strain using a CRISPR/Cas9 genome editing strategy and competed it against a wild-type strain and a strain harboring a synonymous mutation in *CSF1* (*Materials and Methods*; Fig. 3a). We mixed *CSF1*^A2913P^, the synonymous mutant, and the original ancestor in equal proportions and grew these in media with or without 9 µM clotrimazole (Fig. 3a). We found that the *CSF1*^A2913P^ mutation fixed in yeast populations grown in media containing clotrimazole but not in populations grown without clotrimazole, indicating that this mutation improves the fitness of *S. cerevisiae* under the selective pressure of an azole antifungal drug (Fig. 3b).

### Evidence of epistatic interactions between adaptive mutations

The large number of replicates available allowed us to look for patterns of mutation exclusivity and co-occurrence in our evolved clones. These instances can be due to epistatic interactions, which often indicate a functional connection between genes (Lehner 2011; Costanzo *et al.* 2019).

Almost all (91 of 99) clones possessed a mutation in either *PDR1* or *PDR3, which encode paralogous transcription factors that activate the pleiotropic drug response*. We found mutations in these genes in both haploids and diploids, suggesting that they are gain of function (Balzi and Goffeau 1991).

**Fig. 4.**
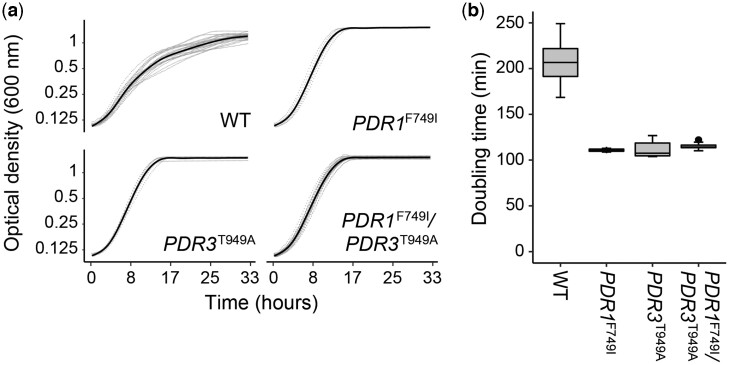
Analysis of growth rates of *PDR1^F749I^* and *PDR3^T949A^* single and double mutants. Haploid *PDR1^F749I^* and *PDR3^T949A^* evolved strains were crossed and sporulated to generate recombinant haploid spores. Spore genotypes were determined by CAPS markers (Supplementary Table 12 in Supplementary File 1, *Materials and Methods*). a) Segregants were arrayed in a 96-well plate and grown in 9 µM clotrimazole media at 30°C in a Biotek Synergy H1 plate reader that measured growth of each strain by optical density (Supplementary Table 13 in Supplementary File 1). b) Doubling time of each genotype was calculated by linear fit of logarithmic growth phase for each curve in a (*Materials and Methods*).

Despite the prevalence of these mutations and the length of our selection protocol, no evolved clone possessed mutations in both *PDR1* and *PDR3*. This may indicate that once a gain-of-function mutation has occurred in one of these paralogs, there is no benefit (or even a negative consequence) to having a second in the context of these experiments. To test this hypothesis, we designed a crossing scheme to generate recombinant progeny in which mutations in both *PDR1* and *PDR3* segregated. To aid in this effort, we examined our list of mutants for clones with (1) opposite mating types, (2) minimal other mutations, and (3) *PDR1* and *PDR3* mutations that could be genotyped by restriction enzyme digestion. Clones Westridge_T_early_1_2017-2018 (*PDR3*^T949A^) and Westridge_S_early_1_2017-2018 (*PDR1*^F749I^ and *HBT1*^T202I^) fit these characteristics. These clones were mated to form a diploid, sporulated, and 16 tetrads were dissected; all segregants were then genotyped and monitored for growth in the presence of 0, 9, and 18 µM clotrimazole (*Materials and Methods*; Fig. 4 and Supplementary Fig. 2). Strains with mutations in both *PDR1* and *PDR3* showed very similar growth rates to strains with a mutation in only one, suggesting that a second mutation does not increase or decrease fitness in the presence (or absence) of clotrimazole. This result may explain the absence of double mutants among sequenced clones.

**Fig. 5.**
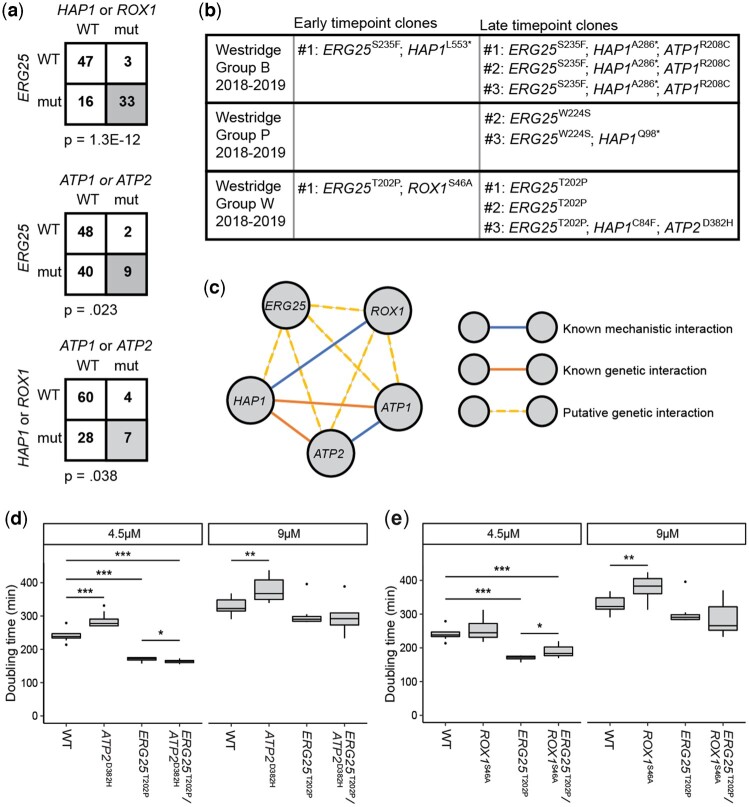
Signatures of epistasis involving *ERG25*. a) Number of clones with genotypes related to the genes *ERG25*, *HAP1*, *ROX1*, *ATP1*, and *ATP2*. Frequency is biased toward double mutant groupings in the bottom right of each square (*P*-values by *t*-test listed below). Clones were sequenced at multiple timepoints; therefore, not all clones represent independent mutation events (*Materials and Methods*). b) Lineages within individual replicates were identified by shared mutations in clones from that replicate. Several lineages are made up of clones with *ERG25* mutations that later acquire mutations in *HAP1*, *ROX1*, *ATP1*, or *ATP2*, as evidenced by multiple individuals with the same *ERG25* mutation but different mutations in *HAP1*, *ROX1*, *ATP1*, or *ATP2*. No lineages were detected in which a *HAP1*, *ROX1*, *ATP1*, or *ATP2* mutant later acquired an *ERG25* mutation. c) Blue lines between *ROX1-HAP1* and *ATP1-ATP2* are known mechanistic relationships (Hap1 regulates Rox1; Atp1 and Atp2 are in the same complex). Orange lines between *HAP1*-*ATP1* and *HAP1*-*ATP2* are previously identified genetic interactions (van Leeuwen *et al.* 2016). The six yellow dashed lines are putative novel genetic interactions supported by presented data. d) Doubling time of segregants from backcrossing of strain containing mutations in *ERG25* and *ATP2* or e) *ERG25* and *ROX1* (*Materials and Methods*) grown in a 96-well plate in 4.5 or 9 *µ*M clotrimazole media at 30°C. Doubling time calculated by linear fit of logarithmic growth phase (Supplementary Fig. S3, *Materials and Methods*). *P*-value by *t*-test, *P < 0.05; **P < 0.01; ***P < 0.001.

We also observed that mutations in *HAP1*, *ROX1*, *ATP1*, and *ATP2* co-occurred with mutations in *ERG25* (Fig. 5a). Specifically, *ERG25* mutants seemed more likely to have secondary mutations in either *HAP1* or *ROX1* and either *ATP1* or *ATP2*. Though approximately half of the sequenced clones have a mutation in *ERG25* (49 of 99), only five mutations in *HAP1*, *ROX1*, *ATP1*, and *ATP2* occurred in an *ERG25* wild-type background, compared to 35 that had a mutation in *ERG25*. In the presence of clotrimazole, *ERG25* mutations generally occurred by early time points, followed by mutations in *HAP1*, *ROX1*, *ATP1*, or *ATP2* which were primarily identified at late time points. This ordering is supported by the higher prevalence of clones with mutations in only *ERG25*, and that clones from lineages with the same *ERG25* mutation have different mutations in *HAP1*, *ROX1*, *ATP1*, or *ATP2* (Fig. 5b). To experimentally test the hypothesis that these combinations are evidence of epistatic interactions, we backcrossed these mutants to a wild-type ancestor to generate panels of mutant strains that possessed combinations of mutations (*Materials and Methods*). We grew these strains in media containing clotrimazole and monitored their growth to calculate a doubling time (Fig. 5, d and e; Supplementary Fig. 3; *Materials and Methods*). Strains with mutations in *ERG25* doubled faster than wild-type strains at a low dose of clotrimazole, but not significantly so at a higher dose. Surprisingly, *ATP2* and *ROX1* single mutants grew more slowly than any other mutant class or the wild-type strains. In addition, mutations in *ATP2* or *ROX1* in combination with *ERG25* mutations did not have a large effect on doubling time compared to *ERG25* single mutants. However, *ATP2* in combination with *ERG25* mutation led to a significantly faster doubling time at a low dose of clotrimazole. Taken together, these results indicate that mutations in *ATP2* and *ROX1* do not provide a direct benefit toward doubling time at the tested concentrations of clotrimazole, and may be selected for a benefit that we did not measure.

## Discussion

In this article, we demonstrated how course-based research experiments with high school students can yield insights into mechanisms of azole resistance. In addition to furthering our science research goals, student involvement advanced their understanding of how genotype and phenotype intersect, which is critical to understanding the process of evolution (NRC 2012; NGSS Lead States 2013). We expand on our pedagogical goals and outcomes from this project in (Taylor 2022).

Laboratory evolution experiments provide time for multiple mutations to occur in a single lineage. This may allow for mutations with smaller or epistatic effects to occur. As evidence of these advantages, all of the novel *CSF1* mutations we identified were found in clones isolated from later time points, as well as much of the evidence implicating P-body mutations and candidate epistatic interactions. Together, our findings underscore the utility of long-term selection to isolate diverse mutations that can impact azole resistance. Increasing the replication of these experiments can improve their power to detect novel adaptive mutations and candidate epistatic interactions, making them ideal for large-scale replication in a classroom setting.

### Concordance with pathogenic isolate sequencing and prior genetic screens

Many of the mutations we identified are in-line with findings from sequencing of drug-resistant pathogenic species of yeast isolated in clinical or agricultural settings (e.g. Ford *et al.* 2015; Paul and Moye-Rowley 2014). Like the clones from our experiments, drug-resistant fungi frequently possess gain-of-function mutations in orthologs of *PDR1* and *PDR3*, copy number variations that lead to increased copy number of the *ERG11* gene, and/or mutations in regulators of ergosterol production such as *UPC2*. CNVs akin to what we observed in *S. cerevisiae* have been shown to impact azole resistance in experimental evolution and clinical isolates of the pathogenic yeast *Candida albicans* (Selmecki *et al.* 2006, 2008, 2009). For instance, aneuploidy has been shown to impact azole resistance in *C. albicans* due to increased copy number of the genes *ERG11* and *TAC1*. Since azoles inhibit the function of *ERG11*, additional copies of this gene may lead to increased expression and thus compensate for the drug’s impact. Clinical and environmental isolates of pathogenic fungi frequently have point mutations in *ERG11* (Fisher *et al.* 2018) that alter the interaction of the enzyme’s active site with azoles, which we did not detect in our experiments.

Surprisingly, we have not observed mutations in *ERG3*, which are frequently identified in pathogenic yeasts with azole resistance. Mutations in *ERG3* are thought to prevent the accumulation of the fungistatic azole intermediate 14 alpha methylergosta 8-24 (28) dienol, produced as the result of Erg11 inhibition by azoles. Instead, we saw mutations in the enzyme Erg25, which is involved downstream of Erg11 in ergosterol biosynthesis. Mutations in Erg25 may act to decrease activity of the enzyme in some way, as decreased expression has been shown to increase azole resistance (Smith *et al.* 2016). Loss of Erg11 and Erg25 function is thought to enable the incorporation of lanosterol as an alternative to ergosterol in the fungal membrane. Similar to Erg3, loss of Erg25 function has been shown to suppress toxicity associated with null mutations in Erg11 or the inhibition of Erg11 by azoles. Importantly, it was shown that reduced heme biosynthesis suppressed this toxicity (Gachotte *et al.* 1997), which is consistent with the appearance of nonfunctional alleles of *HAP1* and *ROX1* that encode regulators of heme biosynthesis.

Another class of known pathogenic mutations that are common among our clones are respiratory deficiencies. Petite mutants of *Candida glabrata* have been isolated in clinical settings (Bouchara *et al.* 2000) and may have increased in vivo virulence (Ferrari *et al.* 2011). These mutants are unable to undergo cellular respiration due to mutations that impact mitochondrial function, which has been shown to increase the activity of Pdr1 and Pdr3 through an as yet unknown post-translational mechanism [Traven *et al.* 2001; possible mechanisms in Shahi *et al.* (2007) and (2010)]. These mutants are thus unable to grow on nonfermentable carbon sources. ρ^0^ (loss of mitochondrial genome) petite mutants were common across the clones we sequenced (72 of 99 clones). The one petite clone from our experiments that still possessed its wild-type mitochondrial genome possessed several mutations, including a nonsynonymous mutation in the transcription factor *TDA9*. *TDA9* regulates acetate production, and *TDA9* null mutants produce less acetic acid during wine fermentation (Walkey *et al.* 2012). *TDA9* has a paralog, *RSF2*, which encodes a gene that regulates growth in glycerol-containing medium (Lu *et al.* 2005). These details make it an attractive candidate for causing the observed petite phenotype.

### The relevance of RAM mutations in azole resistance

*We additionally observed recurrent mutations in genes related to the RAM signaling network.* The RAM network is required for the control of the localization of the transcription factor Ace2 by phosphorylation and also has an important role in polarized growth (Nelson *et al.* 2003). Disruption of RAM network genes results in growth defects and aberrant cell morphologies, as well as sensitivity to cell wall and membrane damaging agents due to changes in cell wall and membrane organization and composition (Nelson *et al.* 2003). Null mutations in proteins of the RAM network cause severe growth defects and lethality in *S. cerevisiae* that is dependent on the genetic background (Xu *et al.* 2019). Literature from other species of yeast indicates that RAM network component knockouts can impact azole resistance in a species or growth phase-dependent manner (Mulhern *et al.* 2006; Walton *et al.* 2006; Song *et al.* 2008; Homann *et al.* 2009; Saputo *et al.* 2012). Surprisingly, in *C. albicans*, null mutations in the RAM network are viable and result in the downregulation of *ERG* genes and hypersusceptibility to azoles (Song *et al.* 2008). All of the RAM network mutations selected by our experiments are either missense or nonsense. The RAM network premature stop codons are all close to the C-terminal end of each protein resulting in truncations that remove less than 10% of the protein (two of six mutations in *TAO3*; one of three in *HYM1*; one of two in *SOG2*). The lack of obvious null mutations suggests that these mutations allow some degree of RAM network functionality. However, we cannot completely rule out secondary site suppressors of lethality, as the RAM mutants appeared late in the evolution experiments in the presence of other mutations. In *CBK1* most missense mutations were identified outside of the conserved AGC kinase domain and known phosphorylation sites (Bidlingmaier *et al.* 2001) and *TAO3* mutations are found at either the far C-terminus (A2283P) or at sites 228 or 229. In both *CBK1* and *TAO3* all mutations overlap with the interaction interfaces that mediate Cbk1 and Tao3 interaction, and C-terminal truncations of Tao3 would also remove a Cbk1-binding site (Nelson *et al.* 2003). This could indicate that evolved mutations are potentially altering the interaction between these RAM network proteins, but how this alteration of RAM network signaling is impacting azole resistance remains to be explored.

### Connection between P-bodies and azole resistance

Three recurrently mutated genes function in an mRNA degradation pathway related to P-bodies. P-bodies have not explicitly been implicated in azole resistance. However, two of our candidate genes (*XRN1* and *DHH1*) have been previously shown to impact azole resistance in separate genome-wide deletion collection screens, and a genome-wide mutagenesis study (Gao *et al.* 2018) found an enrichment for the P-body GO term. It is unclear how these mutations would impact azole resistance at a mechanistic level, though intriguing candidates exist. Evidence suggests that gain-of-function mutations in *CaCDR1* (*PDR1/PDR3* ortholog) impact the stability of its transcripts (Manoharlal *et al.* 2010; Khakhina *et al.* 2018), and that mitochondrial activity regulates these genes through a post-transcriptional mechanism (Shahi *et al.* 2007; Shahi *et al.* 2010). Perturbation of mitochondrial function and application of clotrimazole alters the frequency and morphology of P-bodies (Buchan *et al.* 2011). Loss-of-function mutations in P-body components such as Dhh1, Dcp2, and Xrn1 may then prevent degradation of *PDR1* and *PDR3* transcripts. Such a mechanism would likely have pleiotropic effects on other traits, since P-bodies accumulate many mRNAs.

### Connection between *CSF1* and azole resistance

We observed recurrent mutations in *CSF1*, which encodes a protein of unknown function that localizes to the mitochondria (Reinders *et al.* 2006; Dubreuil *et al.* 2019). Mutations in *CSF1* impact fermentation, specifically at low temperatures (Tokai *et al.* 2000). In a genome-wide screen, *CSF1* was implicated in maturation of glycosylphosphatidylinositol (GPI), a post-translational modification that allows proteins to be targeted to the cell membrane (Čopič *et al.* 2009). Genes involved in this maturation process have been shown to regulate *ERG11* in *C. albicans* and can thus modulate azole resistance (Jain *et al.* 2019). Genome-wide genetic interaction mapping experiments have shown that the interaction profile of *CSF1* is similar to that of many genes involved in cell surface GPI anchor maturation (Supplementary Table 14 in Supplementary File 1; Usaj *et al.* 2017). Moreover, *csf1Δ* has defects in cell wall glycans and is sensitive to membrane and cell wall damaging agents as well as the K1 killer toxin, which is also suggestive of a role in cell wall/membrane integrity (Pagé *et al.* 2003). Furthermore, deletions of *CSF1* have been shown to suppress the azole-resistance phenotype of *erg3Δ* through an unspecified mechanism (Mount 2018). *CSF1* is conserved across many higher eukaryotes. The human gene is hypothesized to be involved in endocytic recycling (Kane *et al.* 2019), and endosomal trafficking mutants have been related to azole resistance in *Candida* (Peters *et al.* 2017). The human homolog is also associated with neonatal death and developmental delay (Kumar *et al.* 2020), so clarifying its mechanism of action and role in cellular function will be of interest.

### Large number of replicates reveals epistatic interactions

We also found novel combinations of mutations that indicate potential epistatic interactions between resistance mutations. Epistatic interactions tend to reflect an underlying mechanistic connection between involved genes (Lehner 2011; Costanzo *et al.* 2019). These interactions can thus provide insight into the basic molecular biology of gene networks involved in drug resistance phenotypes. Knowledge of epistatic interactions can allow researchers to predict genes that are essential for resistance evolution (Lukačišinová *et al.* 2020).

Elucidating the mechanism of the interaction between mutations in *PDR1* and *PDR3* may provide interesting new insights into azole resistance. Single gain-of-function mutations or overexpression of each of these genes individually leads to a dramatic increase in expression of the drug efflux pump *PDR5*, among other resistance factors. It is thus surprising that a second mutation does not provide some additional benefit. This may be due to a ceiling on the benefit possible via these mutations and/or a tradeoff that overrides the beneficial impact of the second mutation, though we did not observe evidence of such a tradeoff when double mutants were grown in media without clotrimazole (Supplementary Fig. 2).

Pdr1 and Pdr3 are paralogs (products of an ancient whole genome duplication) that regulate an overlapping but distinct set of genes related to multidrug resistance and iron metabolism (DeRisi *et al.* 2000; Tuttle *et al.* 2003). They are additionally regulated in overlapping but distinct ways: for instance, loss of mitochondrial genome impacts *PDR3* but not *PDR1* (Hallstrom and Moye-Rowley 2000; Zhang and Moye-Rowley 2001). Many pathogenic yeasts have only a single copy of these transcription factors, but many of the regulatory associations with these genes are conserved (Khakhina *et al.* 2018). Clarifying the mechanism of this epistatic interaction may thus provide insight into resistance mechanisms in pathogenic species. It may additionally provide insight into the forces that shaped the evolution of these paralogs after the ancestral gene was duplicated.

The co-occurrence of mutations in the genes *ERG25, HAP1, ROX1, ATP1*, and *ATP2* suggests that these mutations interact in some way. Based on their frequency and evidence from lineages with identical *ERG25* mutations (Fig. 5b), it is likely that *ERG25* mutations occur first, followed by mutations in *HAP1* or *ROX1* and *ATP1* or *ATP2*. It is thus possible that *ERG25* mutations have a stronger effect on their own, or that mutations in these other genes have a stronger effect when in an *ERG25* background. These points are supported, though not proven, by the finding that individual *ROX1* or *ATP2* mutations seem to lead to a decrease in growth rate, which is not observed in strains that additionally possess mutations in *ERG25* (Fig. 5, d and e).

Hap1 regulates *ROX1* expression (Keng 1992), and they can act as activators or repressors of *ERG* gene expression under different conditions (Serratore *et al.* 2018). *HAP1* mutations have previously been shown to co-occur with mutations in *ATP1* and *ATP2*, and have been speculated to be suppressors of a fitness defect from perturbation of the F1F0 ATP synthase (Leeuwen *et al.* 2016). Together, these observations support that these patterns of co-occurrence are indeed due to epistatic interactions representing an underlying mechanistic connection (Fig. 5c). The mutants isolated from these experiments will be of value for investigating the mechanisms of these interactions. The small effect sizes of *ERG25* mutations, alone or in combination with *ATP2* and *ROX1* mutations (Fig. 5, d and e), suggest that mutations in other genes are important for increased growth rates at higher concentration of azoles; characterizing these relationships could elucidate additional interactions or parallel mechanisms of resistance.

### Effectiveness of yEvo as a scalable course-based research experience

Recent decreases in the cost of whole-genome sequencing have made evolve-and-resequence paradigms more accessible in a broader range of settings. Our findings here demonstrate the power and robustness of this approach in high school classrooms, which could also be applicable to undergraduate courses (Govindan *et al*. 2020). These experiments provide a compelling demonstration of the process of evolution in a pedagogical setting, since they can be explored at many levels of biological organization. Other research areas may also benefit from this type of a student–teacher–scientist partnership in advancing both pedagogical and biomedical research goals.

## Supplementary Material

jkac246_Supplementary_Figure_S1

jkac246_Supplementary_Figure_S2

jkac246_Supplementary_Figure_S3

jkac246_Supplementary_File_S1

## Data Availability

All sequencing data are available at the NCBI Sequence Read Archive (SRA) under BioProject PRJNA742704. Strains and plasmids are available upon request. Supplemental material is available at G3 online.
