# Supplementary figures and images for: yEvo: experimental evolution in high school classrooms selects for novel mutations that impact clotrimazole resistance in Saccharomyces cerevisiae
Source: G3 (Bethesda). 2022 Sep 29;12(11):jkac246. doi: 10.1093/g3journal/jkac246 (PMC9635649; doi:10.1093/g3journal/jkac246)

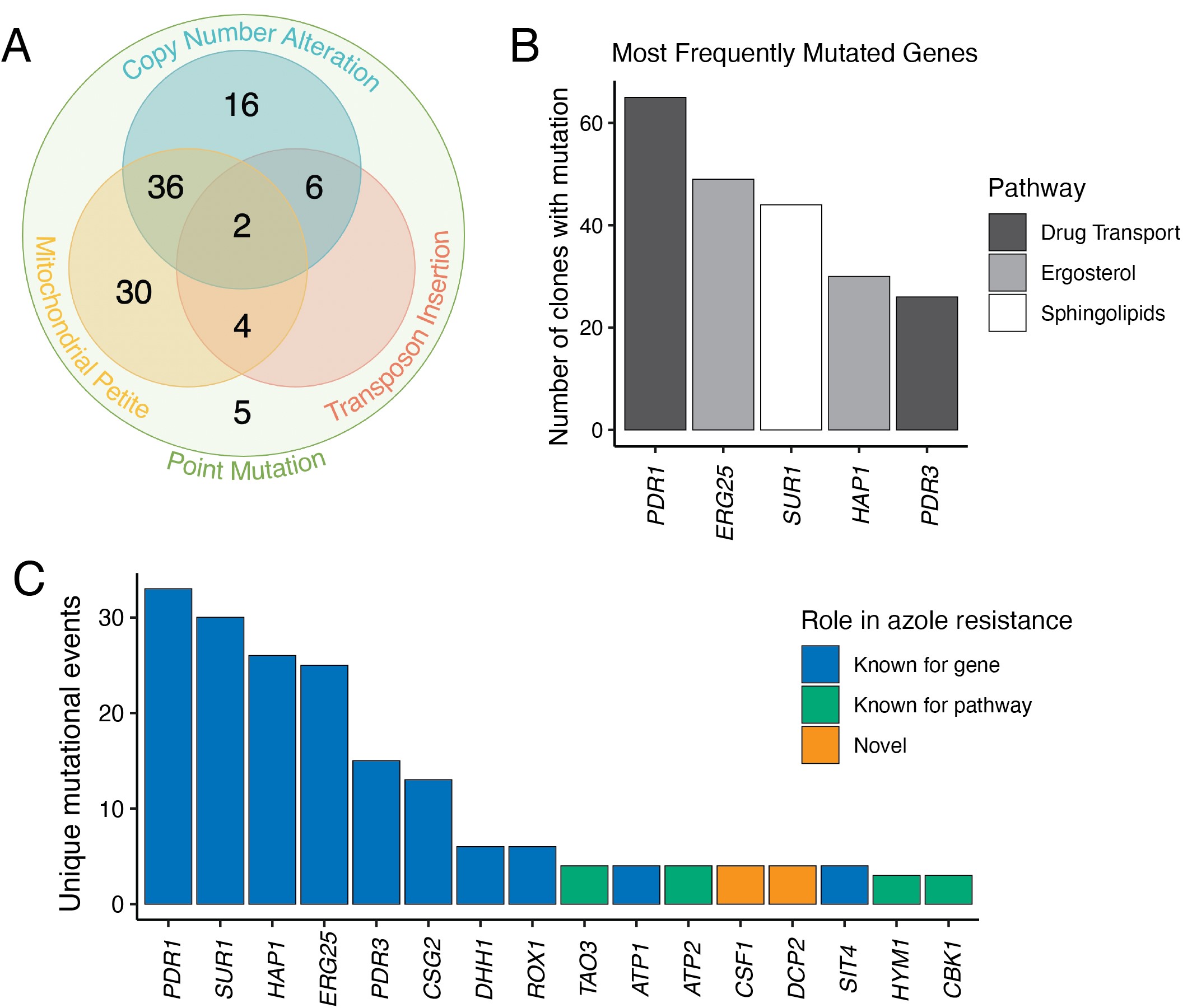

Supplement: jkac246_Supplementary_Figure_S1 [file jkac246_supplementary_figure_s1.jpeg]

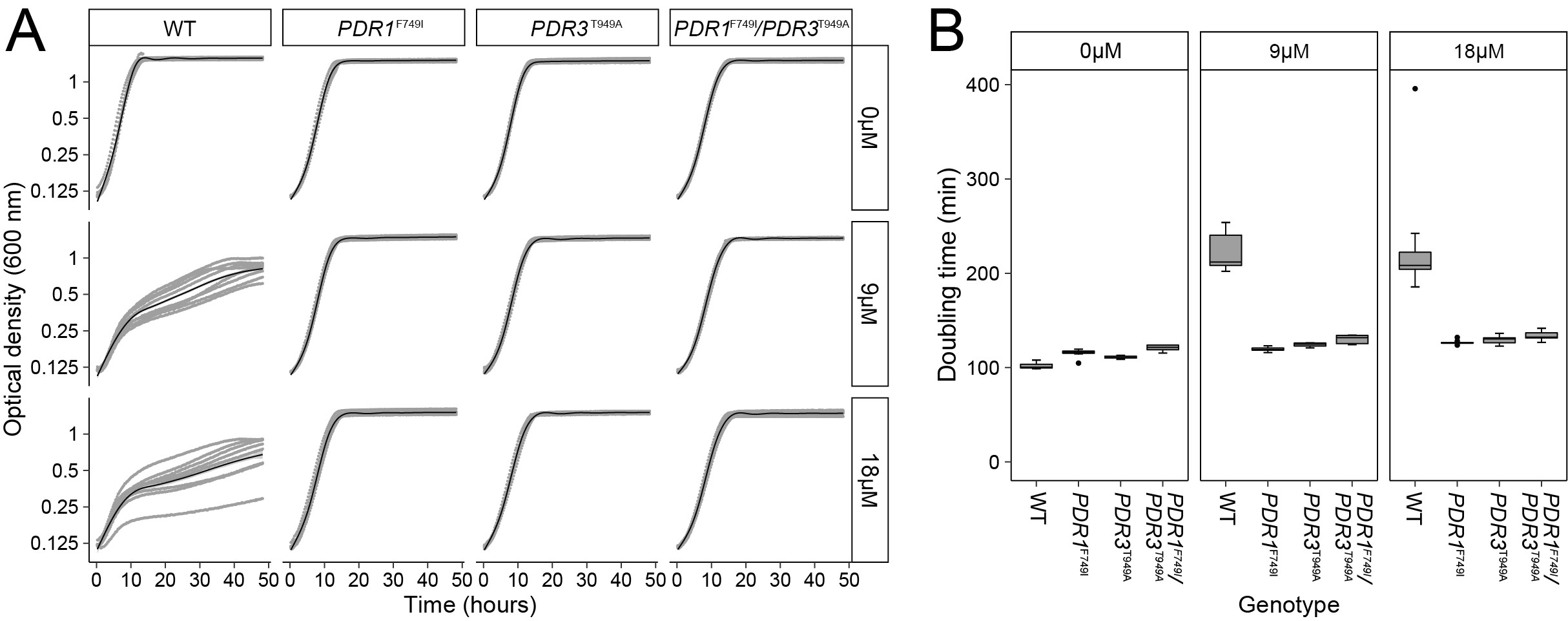

Supplement: jkac246_Supplementary_Figure_S2 [file jkac246_supplementary_figure_s2.jpeg]

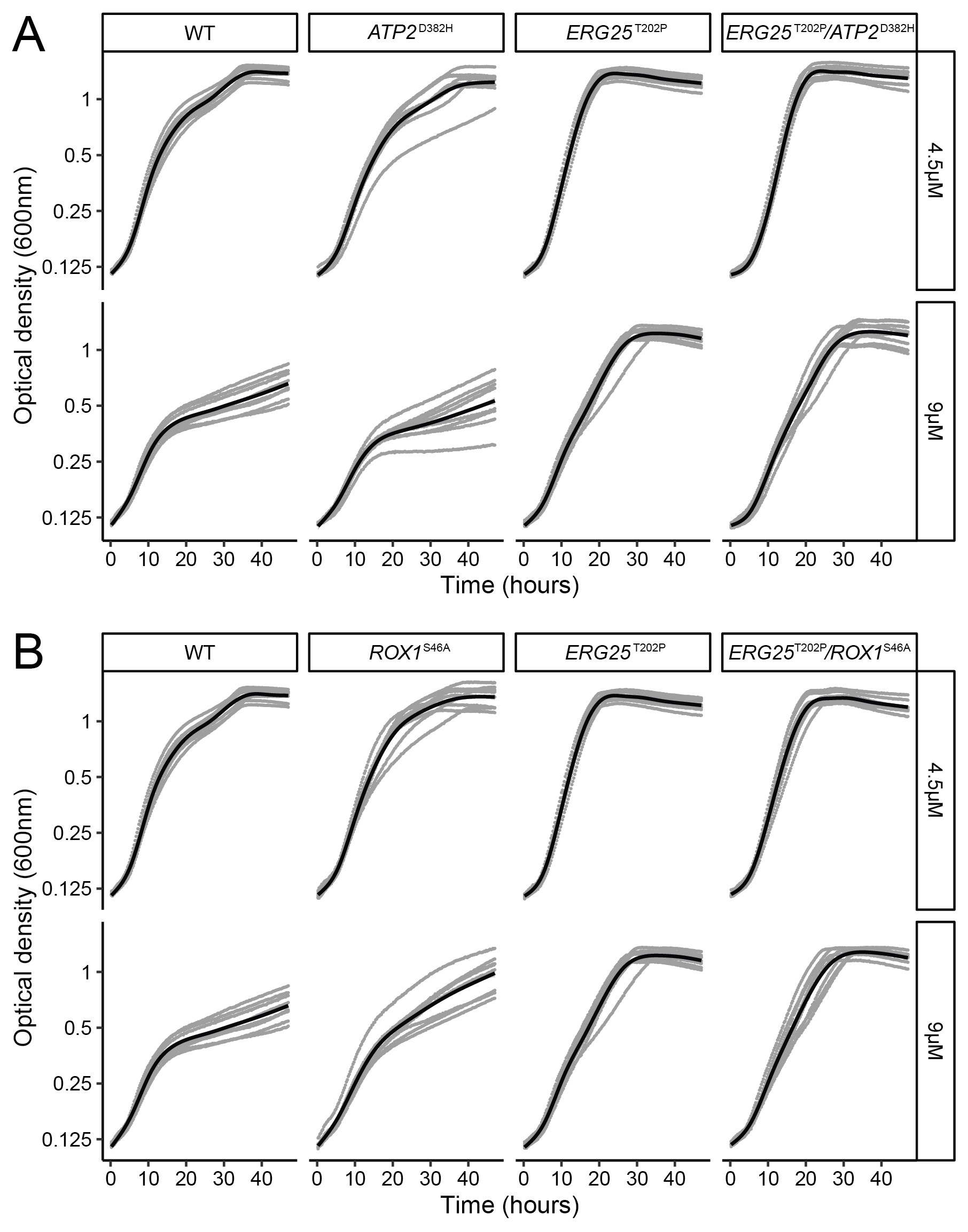

Supplement: jkac246_Supplementary_Figure_S3 [file jkac246_supplementary_figure_s3.jpeg]
